# Supplementary figures and images for: YAP1 activation by human papillomavirus E7 promotes basal cell identity in squamous epithelia
Source: eLife. 2022 Feb 16;11:e75466. doi: 10.7554/eLife.75466 (PMC8959598; doi:10.7554/eLife.75466)

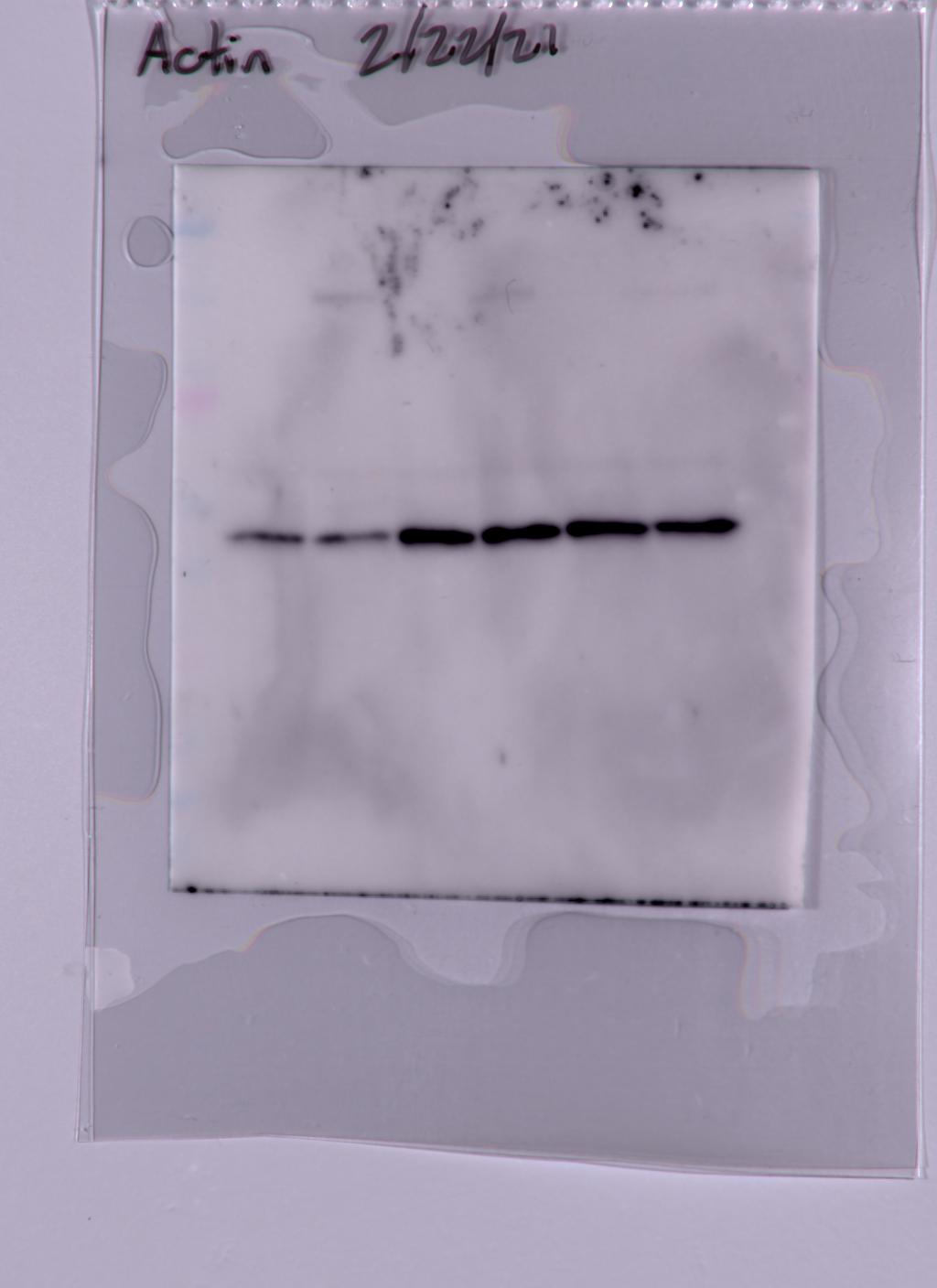

Supplement: Figure 4—source data 1. — Original uncropped images from YAP1, TAZ, V5, and actin Western blots presented in Figure 4B are included as individual image files and as a compiled summary document. [file elife-75466-fig4-data1.zip › Figure 4B/Figure 4B Actin.jpg]

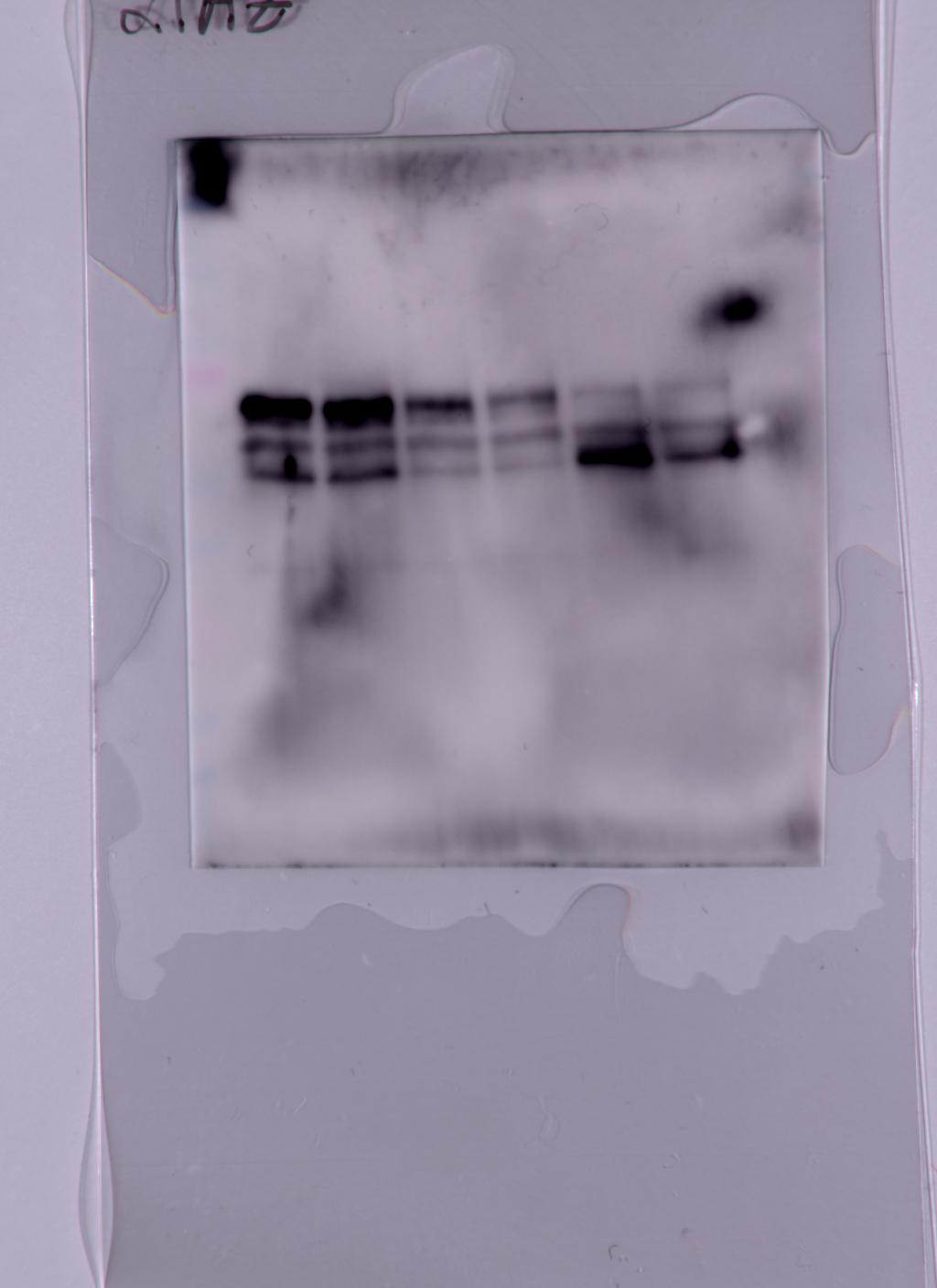

Supplement: Figure 4—source data 1. — Original uncropped images from YAP1, TAZ, V5, and actin Western blots presented in Figure 4B are included as individual image files and as a compiled summary document. [file elife-75466-fig4-data1.zip › Figure 4B/Figure 4B TAZ.jpg]

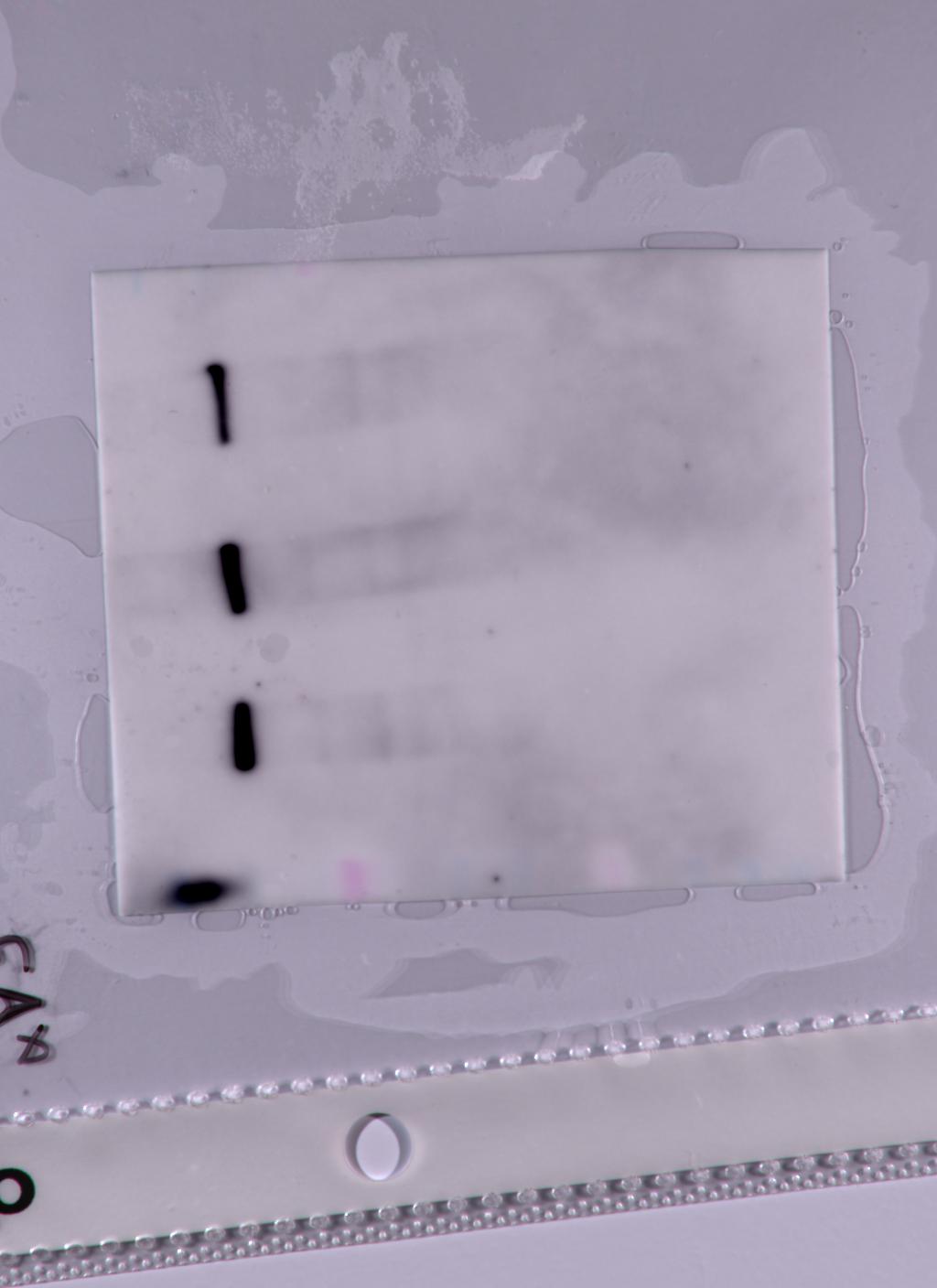

Supplement: Figure 4—source data 1. — Original uncropped images from YAP1, TAZ, V5, and actin Western blots presented in Figure 4B are included as individual image files and as a compiled summary document. [file elife-75466-fig4-data1.zip › Figure 4B/Figure 4B V5.jpg]

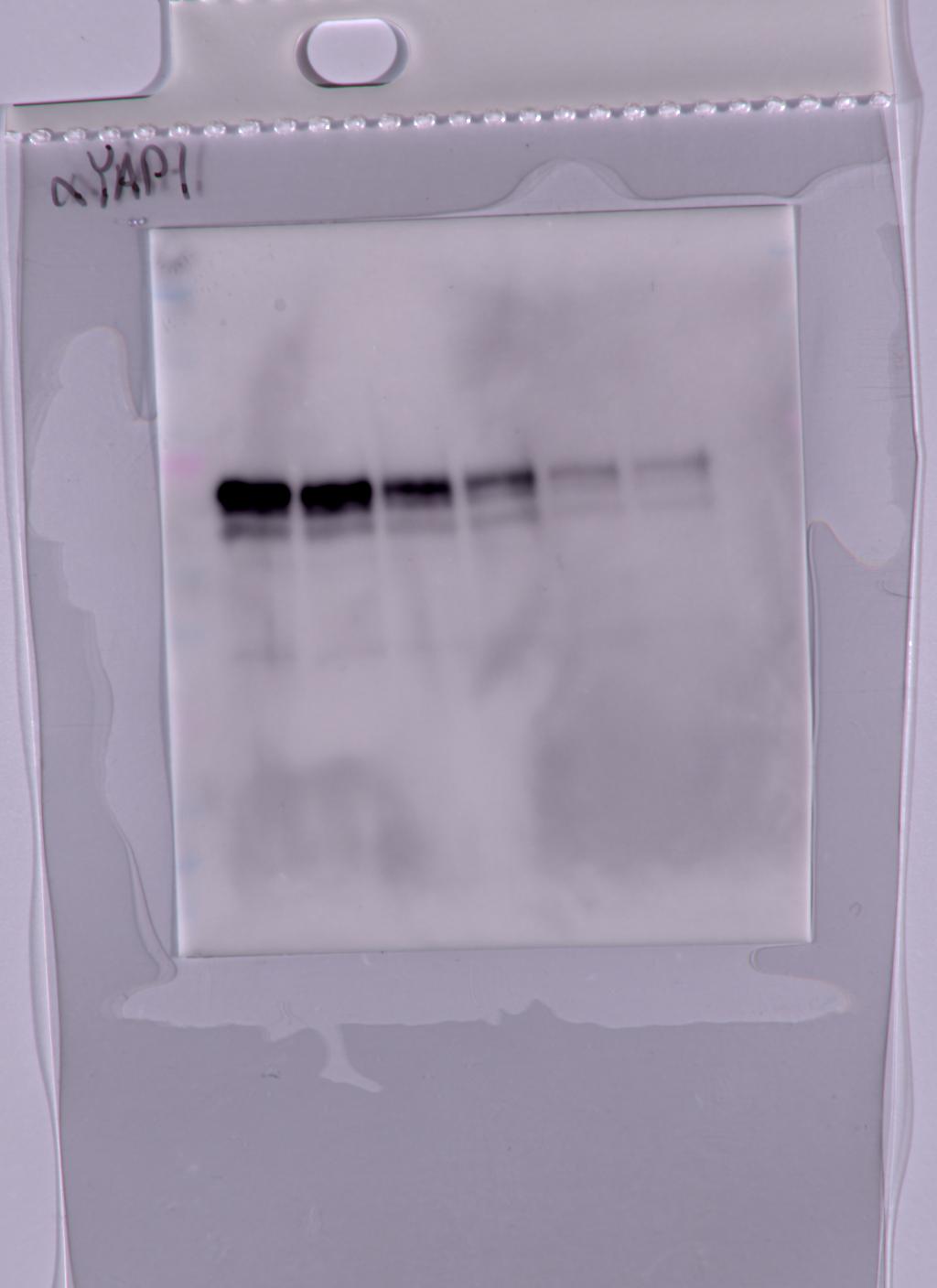

Supplement: Figure 4—source data 1. — Original uncropped images from YAP1, TAZ, V5, and actin Western blots presented in Figure 4B are included as individual image files and as a compiled summary document. [file elife-75466-fig4-data1.zip › Figure 4B/FIgure 4B YAP1.jpg]

**Figure 4B**

anti-V5

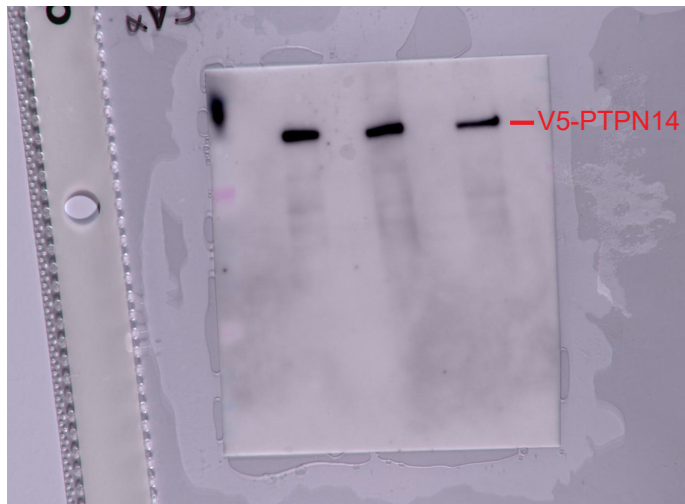

anti-YAP1

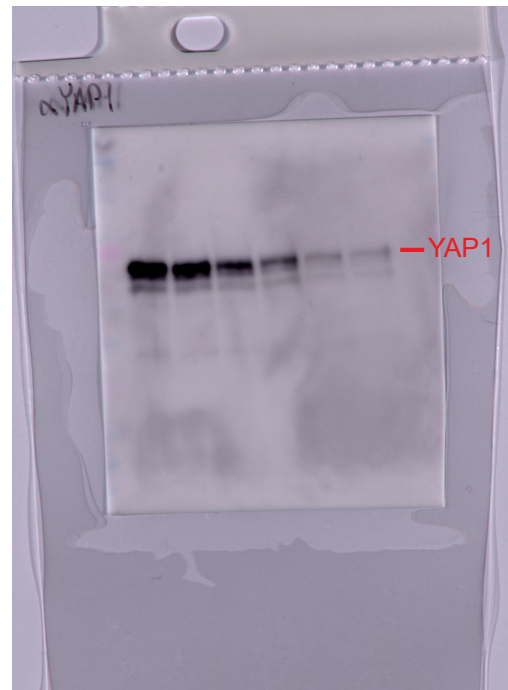

anti-TAZ

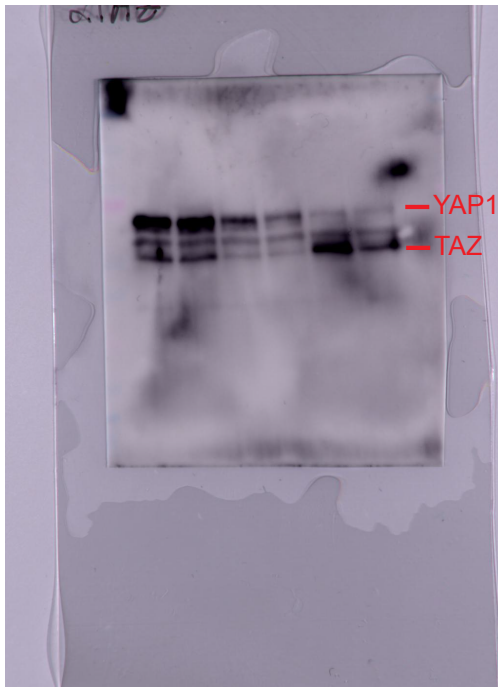

anti-Actin

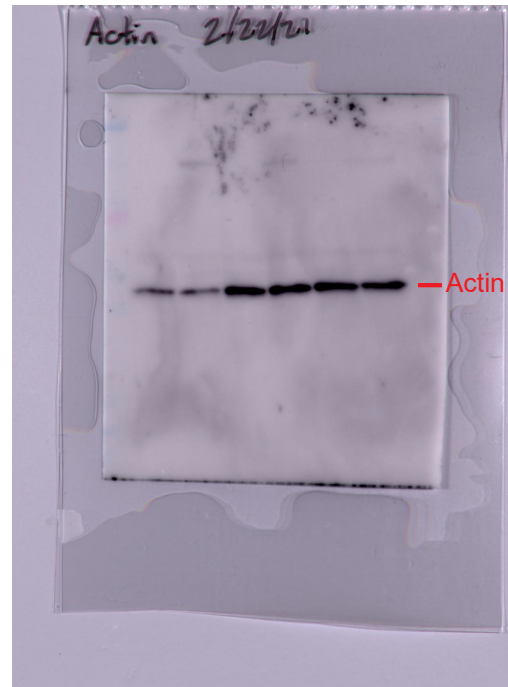

Supplement: Figure 4—source data 1. — Original uncropped images from YAP1, TAZ, V5, and actin Western blots presented in Figure 4B are included as individual image files and as a compiled summary document. [file elife-75466-fig4-data1.zip › Figure 4B/Figure 4B.pdf]

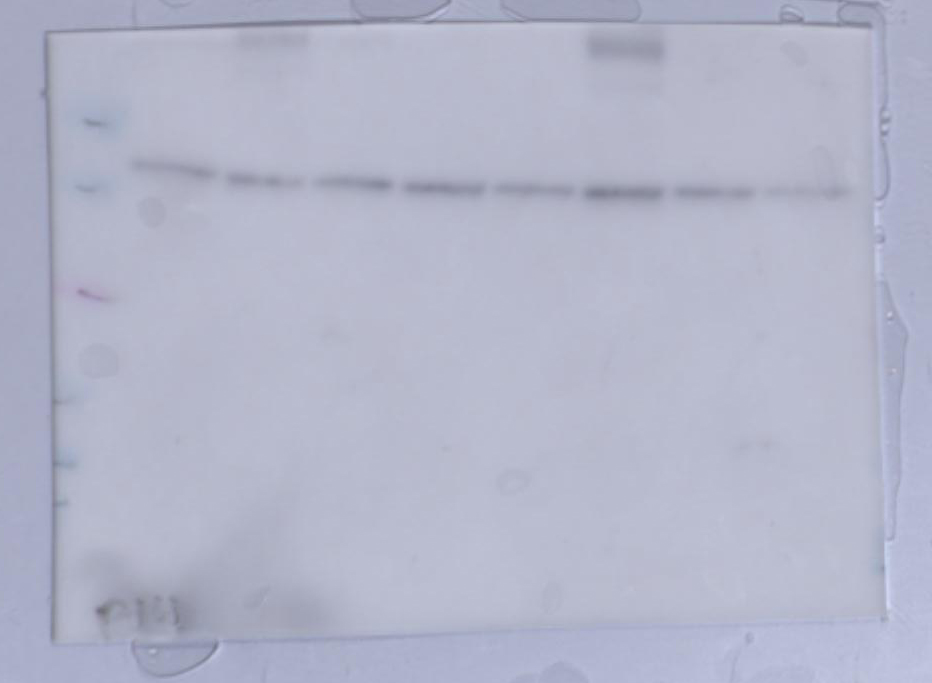

Supplement: Figure 4—figure supplement 1—source data 1. — Original uncropped images from PTPN14, involucrin, V5, and actin Western blots presented in Figure 4—figure supplement 1A are included as individual image files (cropped to individual gels and uncropped versions) and as a compiled summary document. [file elife-75466-fig4-figsupp1-data1.zip › Figure 4--FS1A/cropped to individual gels/Figure 4--FS1A Actin.jpg]

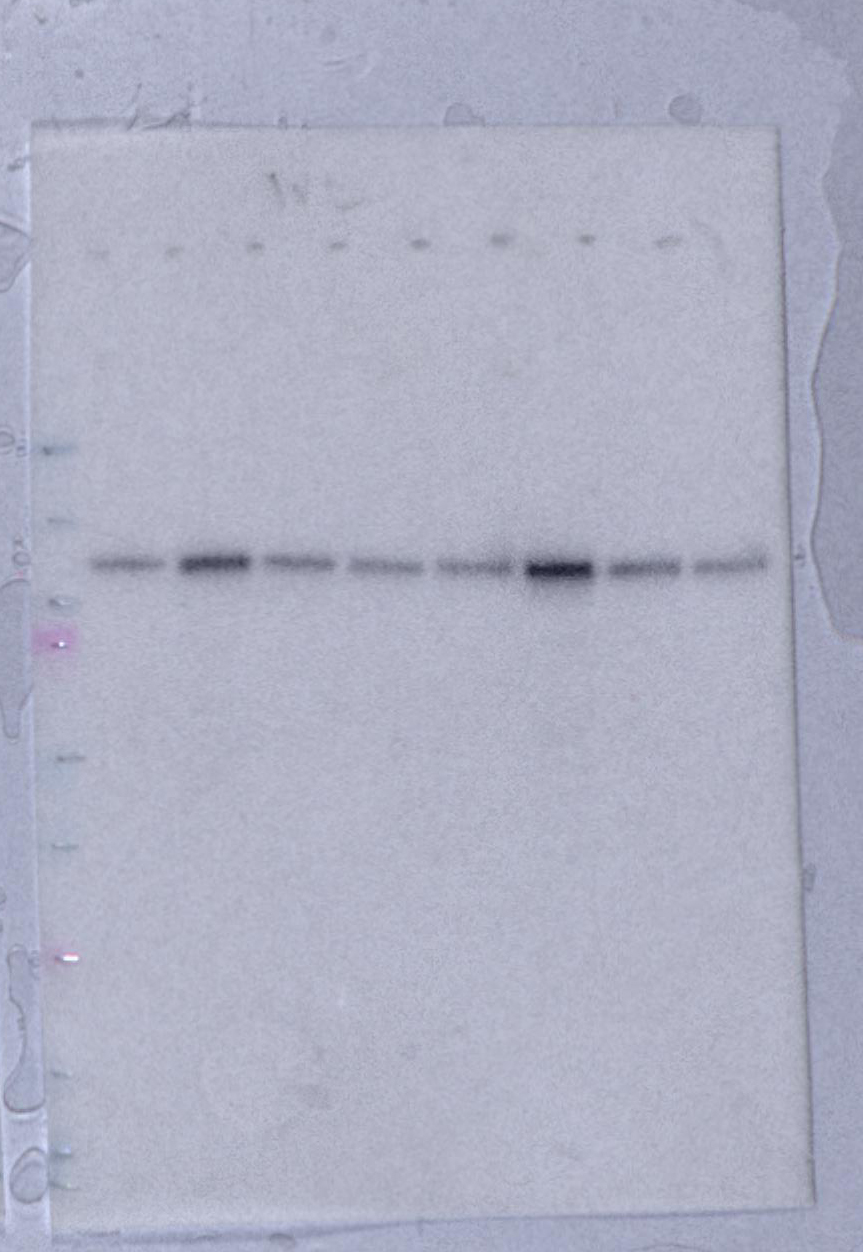

Supplement: Figure 4—figure supplement 1—source data 1. — Original uncropped images from PTPN14, involucrin, V5, and actin Western blots presented in Figure 4—figure supplement 1A are included as individual image files (cropped to individual gels and uncropped versions) and as a compiled summary document. [file elife-75466-fig4-figsupp1-data1.zip › Figure 4--FS1A/cropped to individual gels/Figure 4--FS1A IVL.jpg]

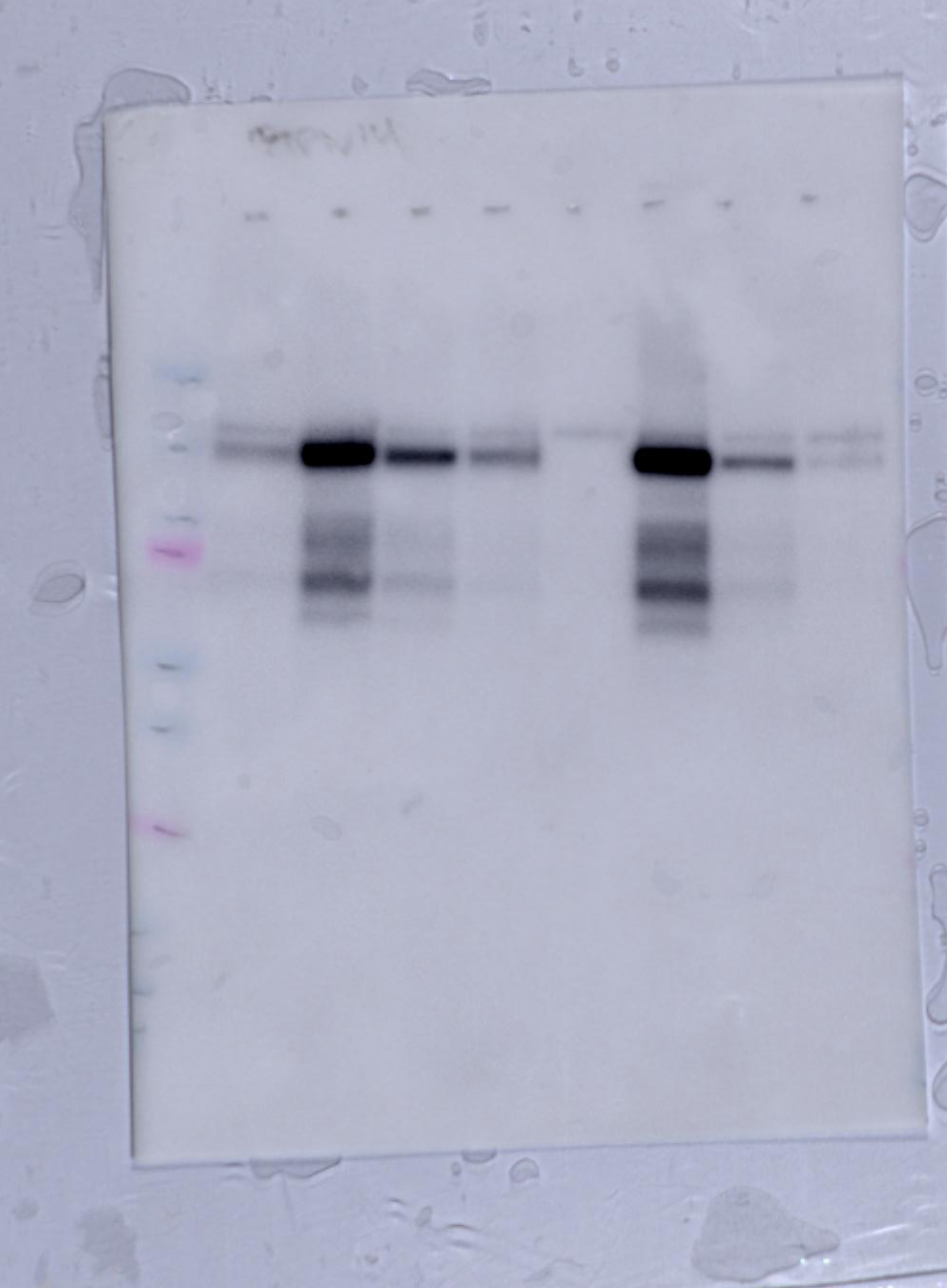

Supplement: Figure 4—figure supplement 1—source data 1. — Original uncropped images from PTPN14, involucrin, V5, and actin Western blots presented in Figure 4—figure supplement 1A are included as individual image files (cropped to individual gels and uncropped versions) and as a compiled summary document. [file elife-75466-fig4-figsupp1-data1.zip › Figure 4--FS1A/cropped to individual gels/Figure 4--FS1A PTPN14.jpg]

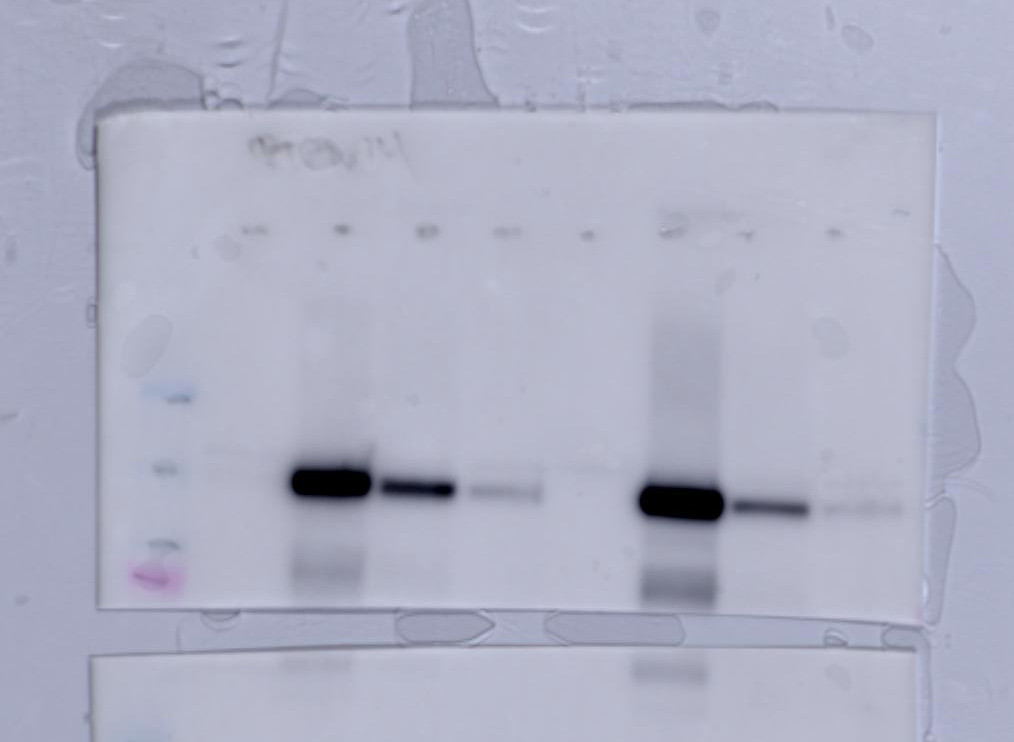

Supplement: Figure 4—figure supplement 1—source data 1. — Original uncropped images from PTPN14, involucrin, V5, and actin Western blots presented in Figure 4—figure supplement 1A are included as individual image files (cropped to individual gels and uncropped versions) and as a compiled summary document. [file elife-75466-fig4-figsupp1-data1.zip › Figure 4--FS1A/cropped to individual gels/Figure 4--FS1A V5.jpg]

# Figure 4-figure supplement 1A

anti-PTPN14

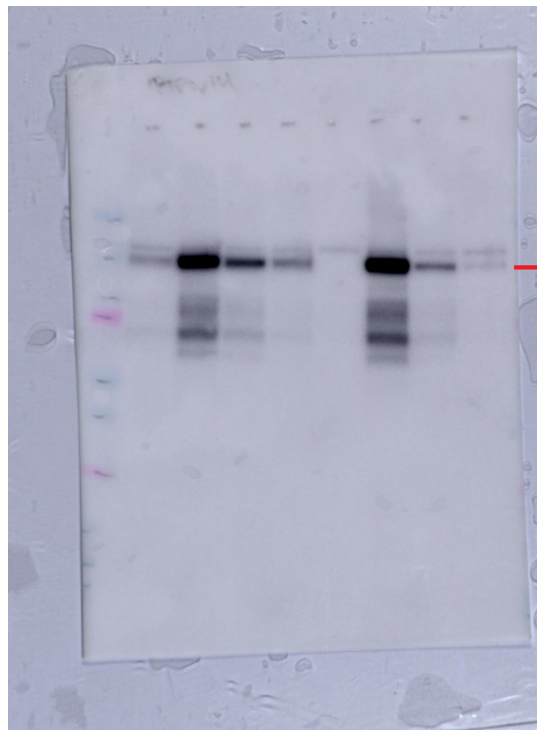

anti-V5

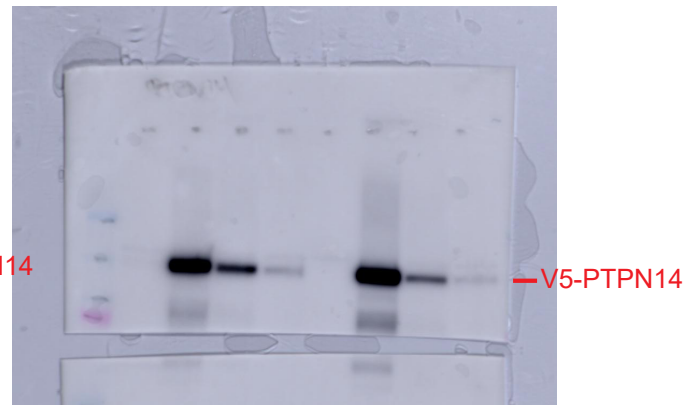

anti-IVL

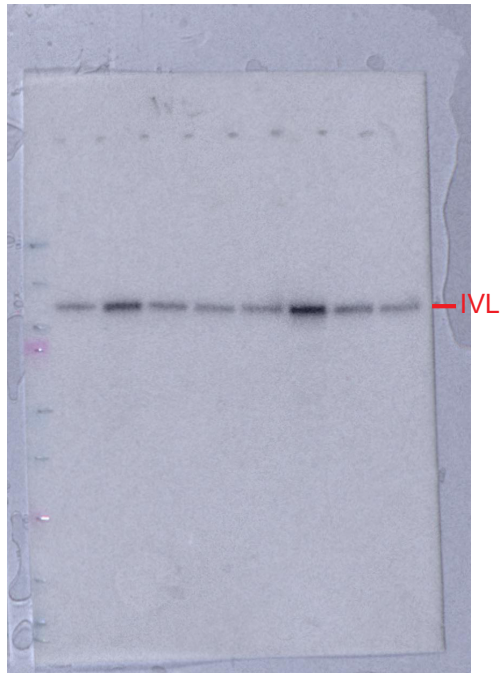

anti-Actin

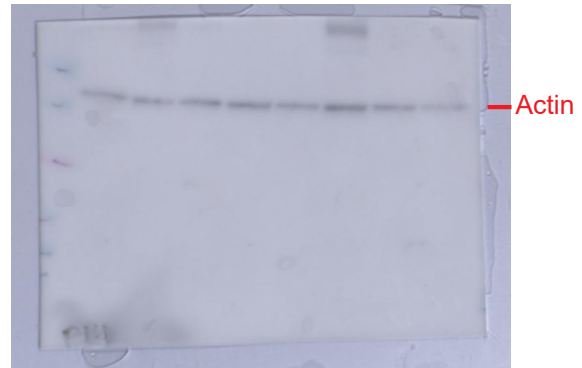

Supplement: Figure 4—figure supplement 1—source data 1. — Original uncropped images from PTPN14, involucrin, V5, and actin Western blots presented in Figure 4—figure supplement 1A are included as individual image files (cropped to individual gels and uncropped versions) and as a compiled summary document. [file elife-75466-fig4-figsupp1-data1.zip › Figure 4--FS1A/Figure 4--FS1A.pdf]

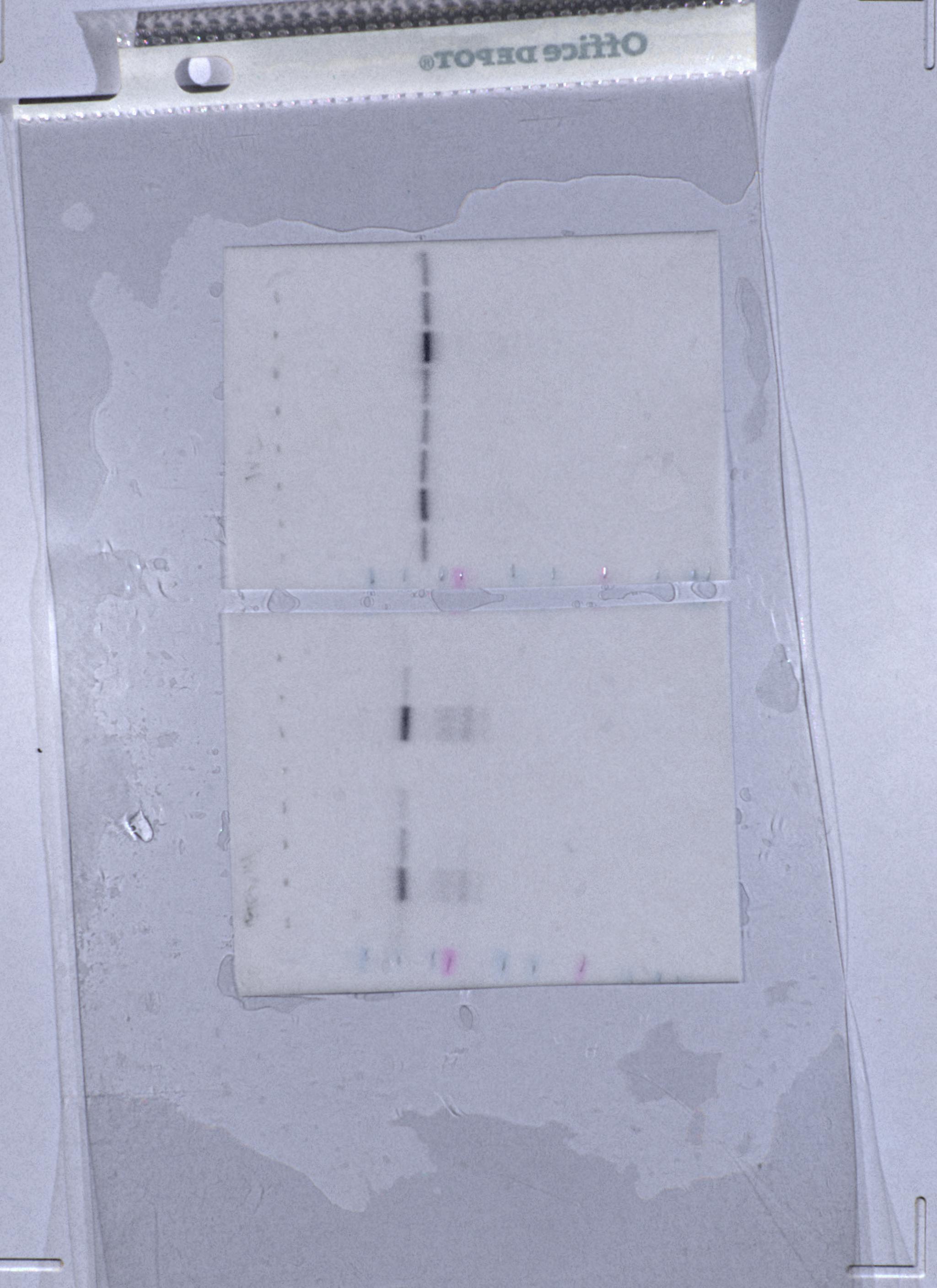

Supplement: Figure 4—figure supplement 1—source data 1. — Original uncropped images from PTPN14, involucrin, V5, and actin Western blots presented in Figure 4—figure supplement 1A are included as individual image files (cropped to individual gels and uncropped versions) and as a compiled summary document. [file elife-75466-fig4-figsupp1-data1.zip › Figure 4--FS1A/uncropped/Figure 4--FS1A PTPN14 and IVL (Exposure for IVL).jpg]

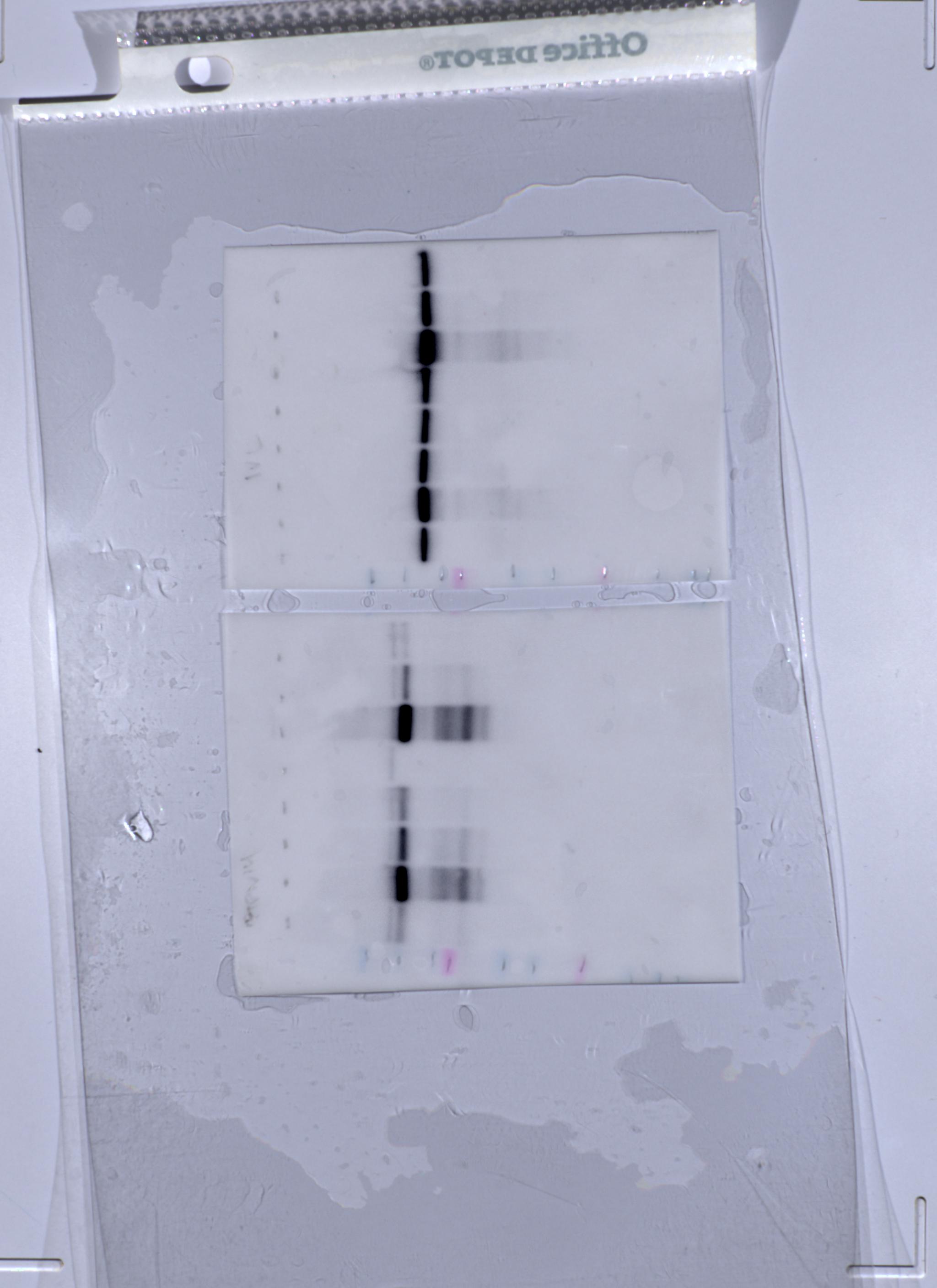

Supplement: Figure 4—figure supplement 1—source data 1. — Original uncropped images from PTPN14, involucrin, V5, and actin Western blots presented in Figure 4—figure supplement 1A are included as individual image files (cropped to individual gels and uncropped versions) and as a compiled summary document. [file elife-75466-fig4-figsupp1-data1.zip › Figure 4--FS1A/uncropped/Figure 4--FS1A PTPN14 and IVL (Exposure for PTPN14).jpg]

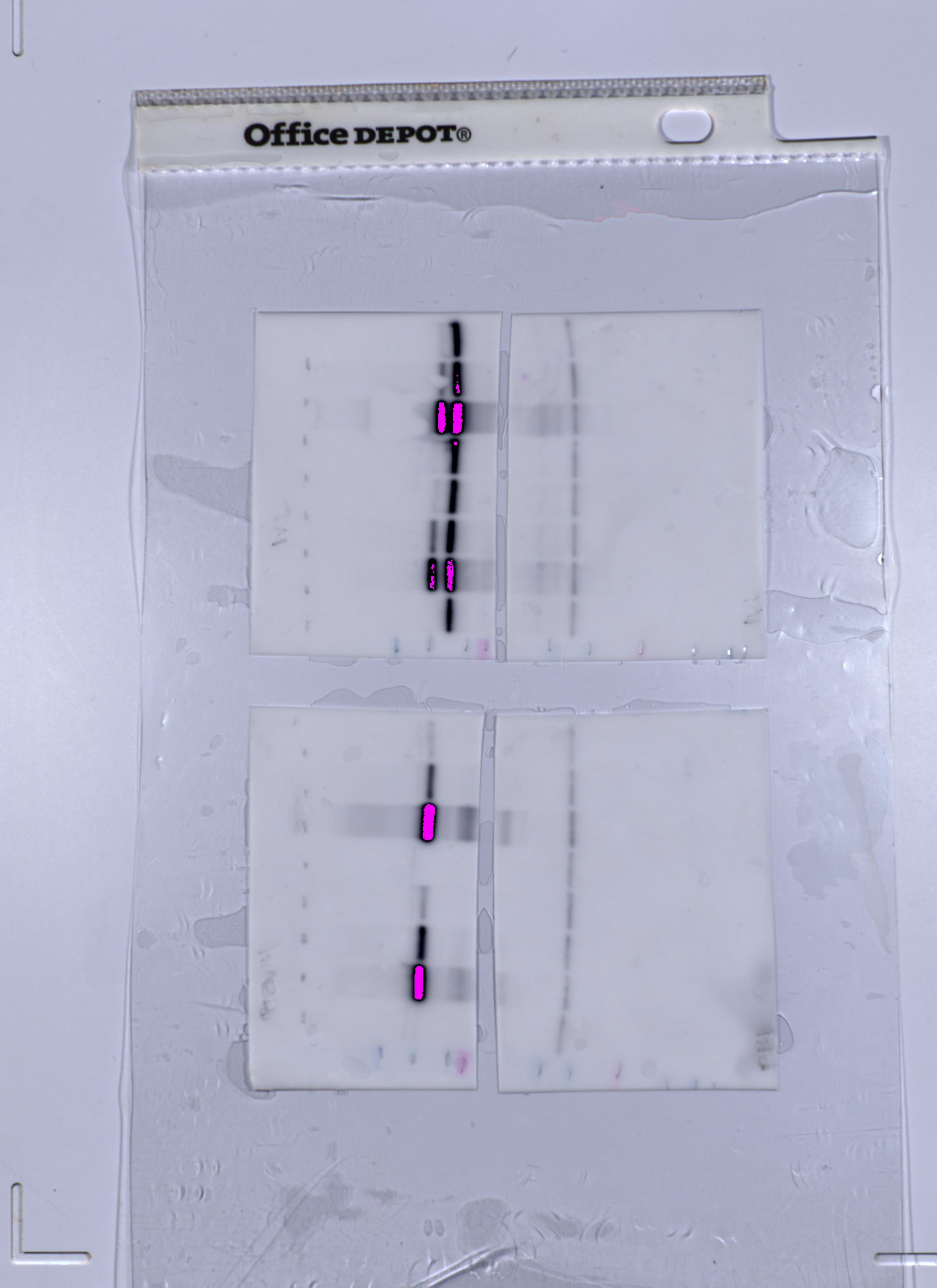

Supplement: Figure 4—figure supplement 1—source data 1. — Original uncropped images from PTPN14, involucrin, V5, and actin Western blots presented in Figure 4—figure supplement 1A are included as individual image files (cropped to individual gels and uncropped versions) and as a compiled summary document. [file elife-75466-fig4-figsupp1-data1.zip › Figure 4--FS1A/uncropped/Figure 4--FS1A V5 and Actin (Exposure for Actin).jpg]

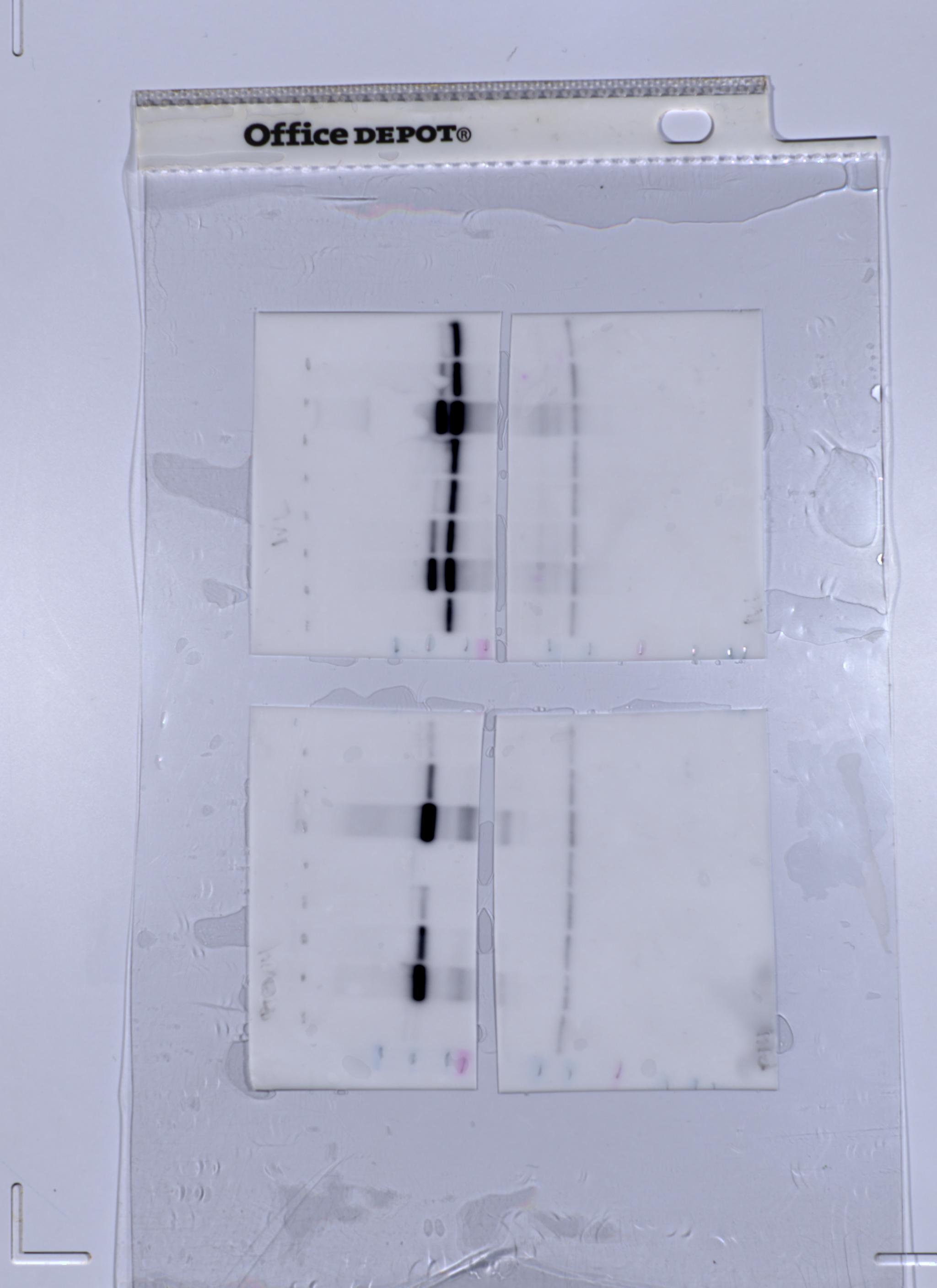

Supplement: Figure 4—figure supplement 1—source data 1. — Original uncropped images from PTPN14, involucrin, V5, and actin Western blots presented in Figure 4—figure supplement 1A are included as individual image files (cropped to individual gels and uncropped versions) and as a compiled summary document. [file elife-75466-fig4-figsupp1-data1.zip › Figure 4--FS1A/uncropped/Figure 4--FS1A V5 and Actin (Exposure for V5).jpg]

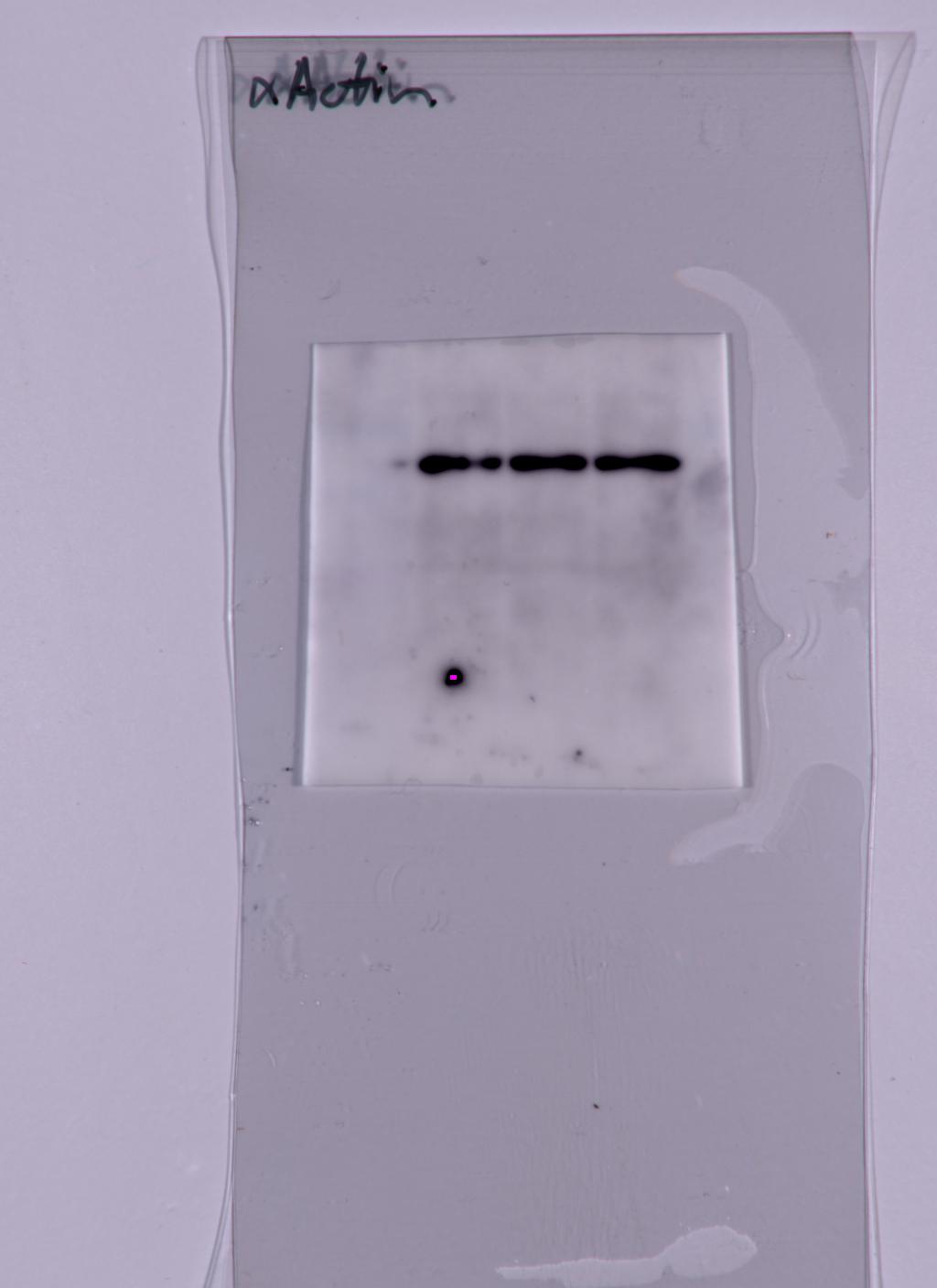

Supplement: Figure 7—figure supplement 1—source data 1. — Original uncropped images from PTPN14 and actin Western blots presented in Figure 7—figure supplement 1D are included as individual image files and as a compiled summary document. [file elife-75466-fig7-figsupp1-data1.zip › Figure 7--FS1D/Figure 7--FS1D Actin.jpg]

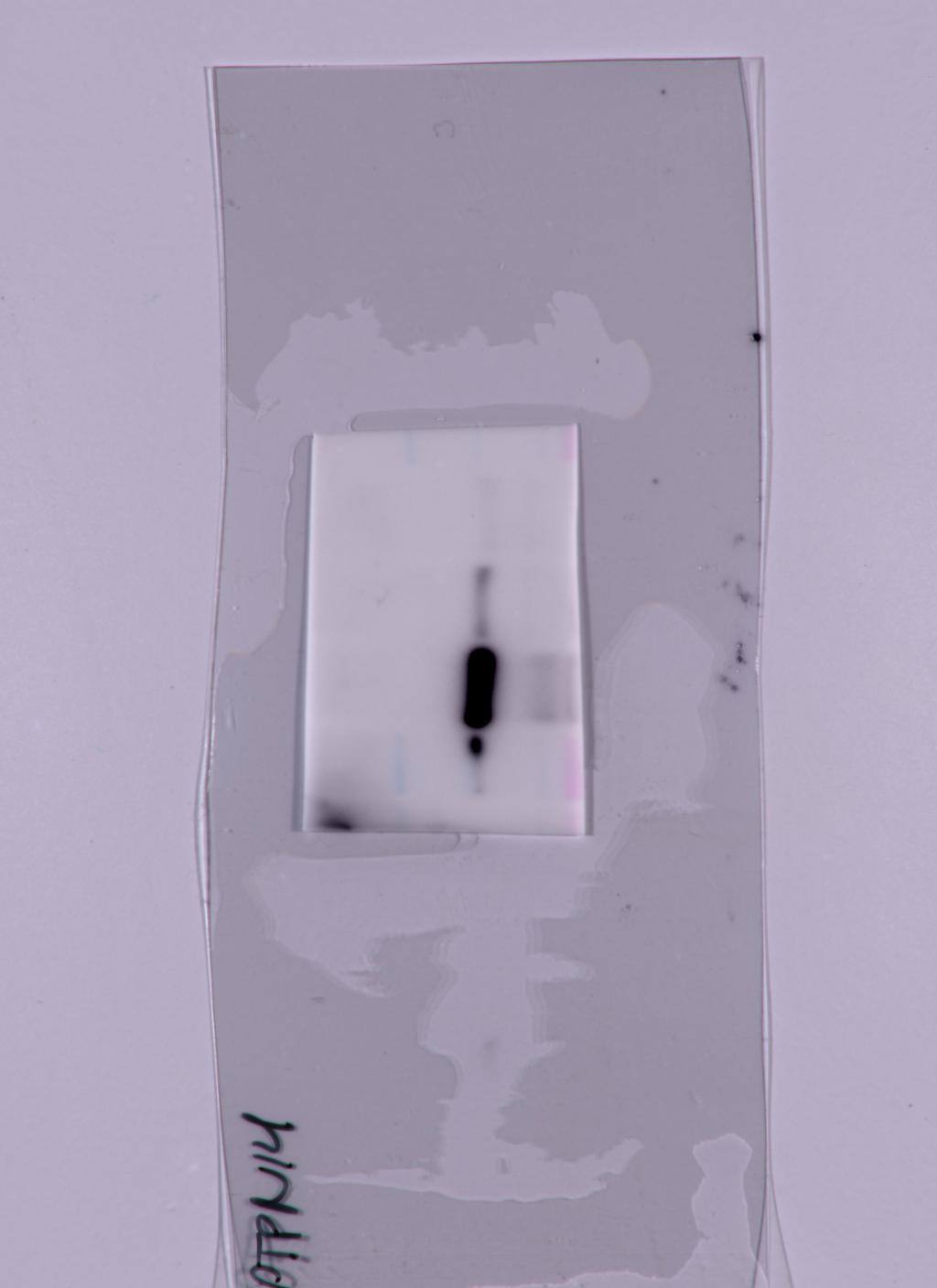

Supplement: Figure 7—figure supplement 1—source data 1. — Original uncropped images from PTPN14 and actin Western blots presented in Figure 7—figure supplement 1D are included as individual image files and as a compiled summary document. [file elife-75466-fig7-figsupp1-data1.zip › Figure 7--FS1D/Figure 7--FS1D PTPN14.jpg]

## Figure 7-figure supplement 1D

anti-PTPN14

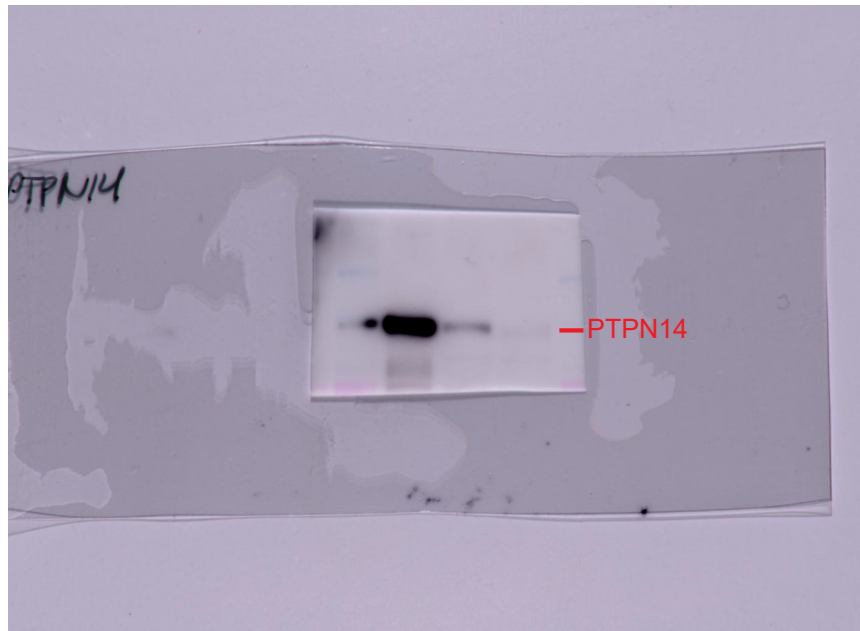

anti-Actin

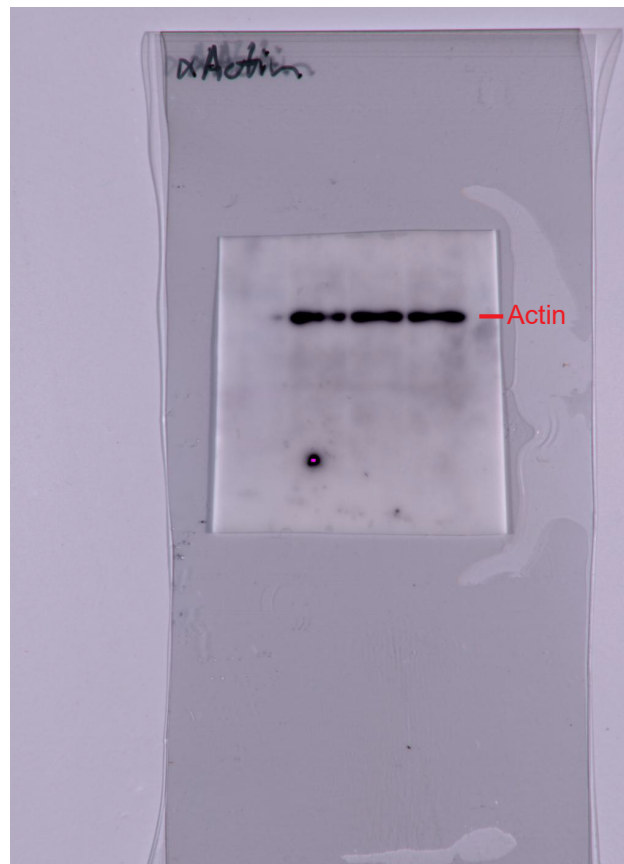

Supplement: Figure 7—figure supplement 1—source data 1. — Original uncropped images from PTPN14 and actin Western blots presented in Figure 7—figure supplement 1D are included as individual image files and as a compiled summary document. [file elife-75466-fig7-figsupp1-data1.zip › Figure 7--FS1D/Figure 7--FS1D.pdf]

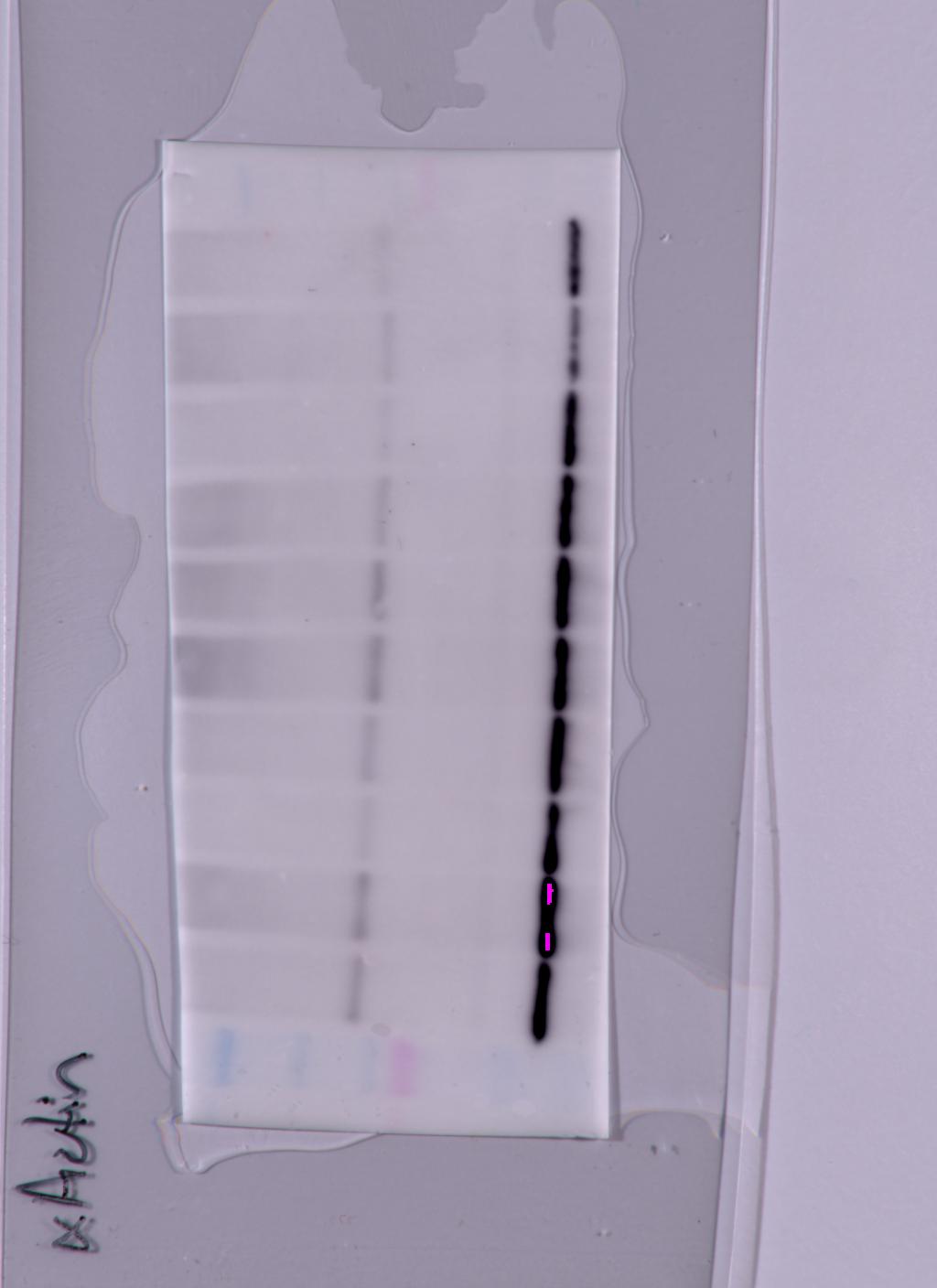

Supplement: Figure 7—figure supplement 1—source data 2. — Original uncropped images from PTPN14, RB1, and actin Western blots presented in Figure 7—figure supplement 1F are included as individual image files and as a compiled summary document. [file elife-75466-fig7-figsupp1-data2.zip › Figure 7--FS1F/Figure 7--FS1F Actin.jpg]

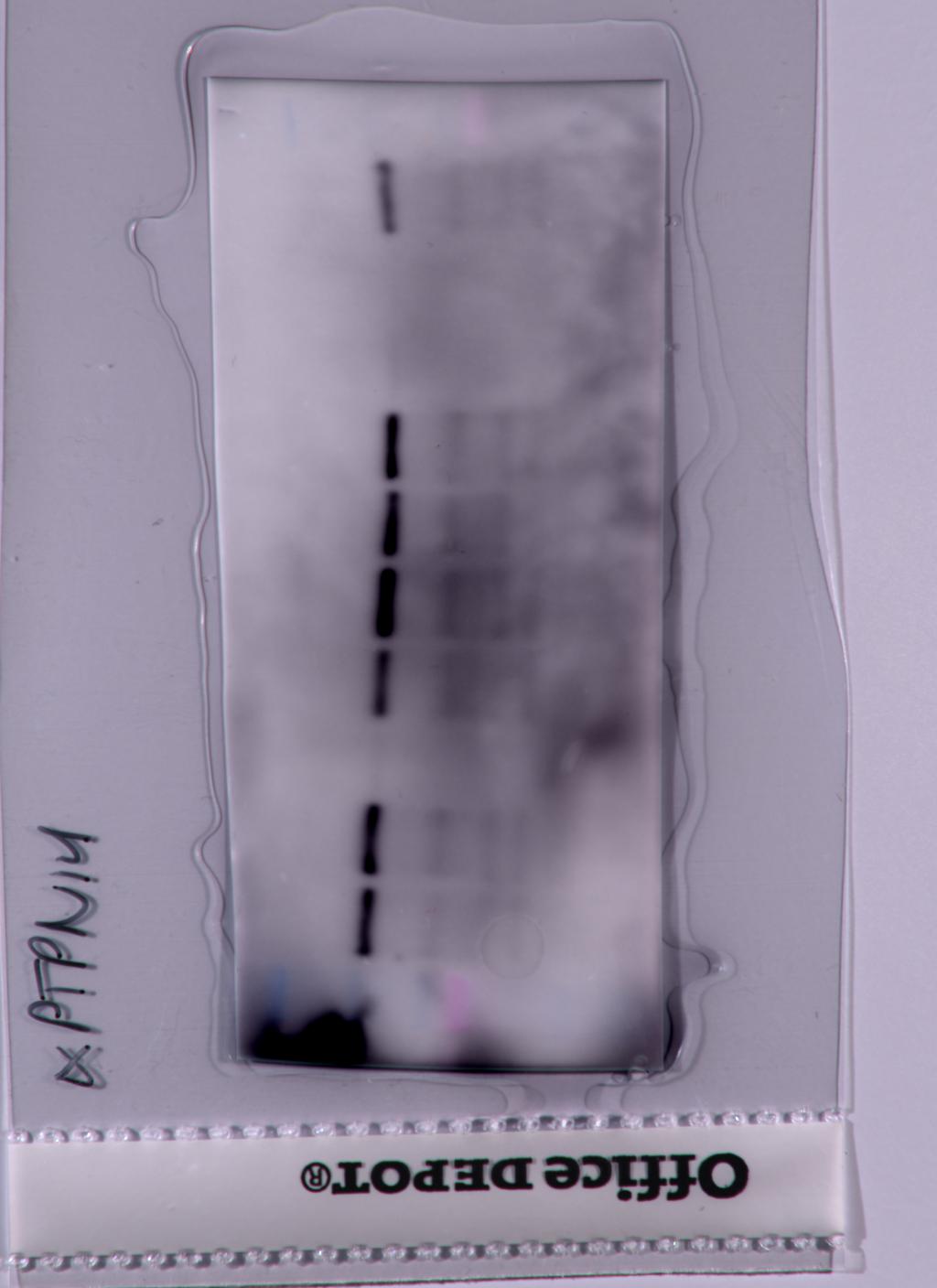

Supplement: Figure 7—figure supplement 1—source data 2. — Original uncropped images from PTPN14, RB1, and actin Western blots presented in Figure 7—figure supplement 1F are included as individual image files and as a compiled summary document. [file elife-75466-fig7-figsupp1-data2.zip › Figure 7--FS1F/FIgure 7--FS1F PTPN14.jpg]

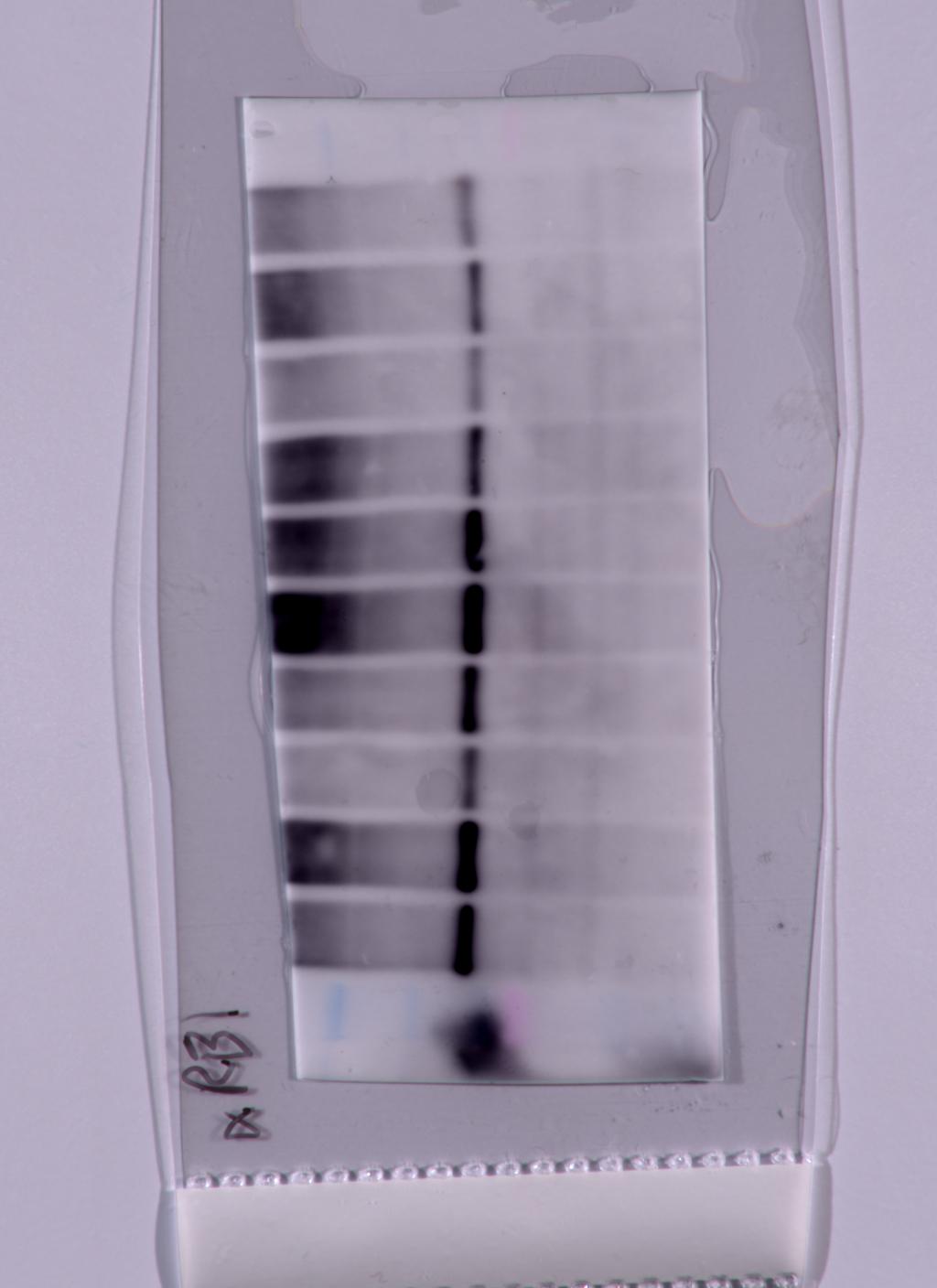

Supplement: Figure 7—figure supplement 1—source data 2. — Original uncropped images from PTPN14, RB1, and actin Western blots presented in Figure 7—figure supplement 1F are included as individual image files and as a compiled summary document. [file elife-75466-fig7-figsupp1-data2.zip › Figure 7--FS1F/Figure 7--FS1F RB1.jpg]

**Figure 7-figure supplement 1F**

anti-PTPN14

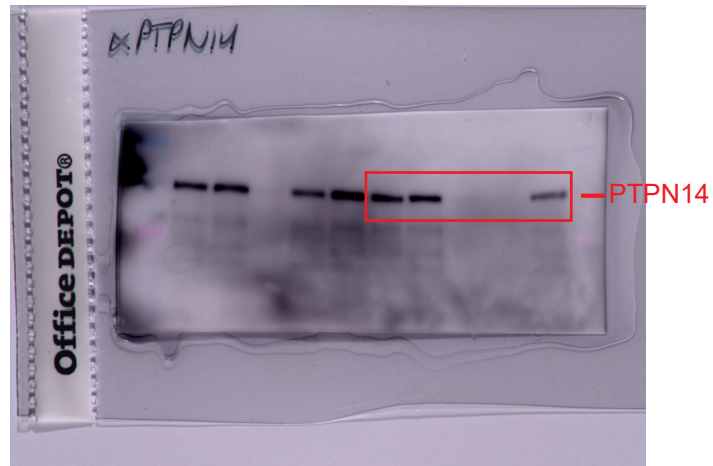

anti-RB1

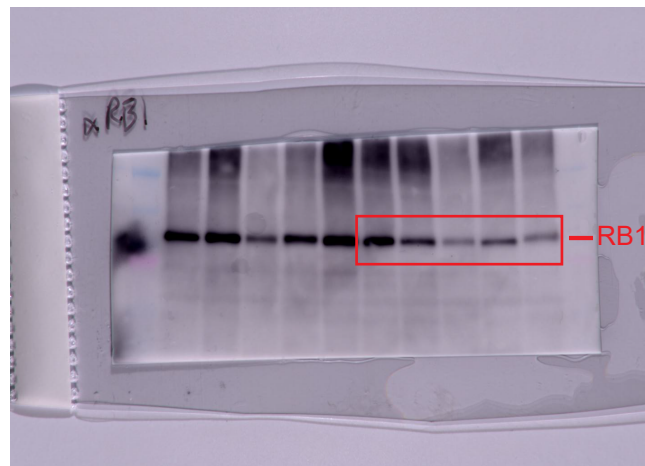

anti-Actin

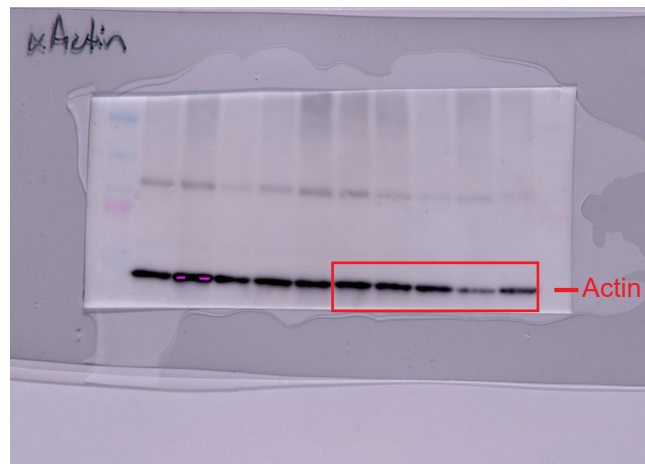

Supplement: Figure 7—figure supplement 1—source data 2. — Original uncropped images from PTPN14, RB1, and actin Western blots presented in Figure 7—figure supplement 1F are included as individual image files and as a compiled summary document. [file elife-75466-fig7-figsupp1-data2.zip › Figure 7--FS1F/Figure 7--FS1F.pdf]
